# Age-specific prevalence of the metabolic syndrome defined by the International Diabetes Federation and the National Cholesterol Education Program: the Norwegian HUNT 2 study

**DOI:** 10.1186/1471-2458-7-220

**Published:** 2007-08-29

**Authors:** Bjørn Hildrum, Arnstein Mykletun, Torstein Hole, Kristian Midthjell, Alv A Dahl

**Affiliations:** 1 Department of Psychiatry, Namsos Hospital, Namsos, Norway; 2 Norwegian University of Science and Technology, Trondheim, Norway; 3 Research Centre for Health Promotion, University of Bergen, Bergen, Norway; 4 National Institute of Public Health, Division Epidemiology, Department of Mental Health, Oslo, Norway; 5 Section of Cardiology, Department of Internal Medicine, Ålesund Hospital, Ålesund, Norway; 6 HUNT Research Centre, Department of Public Health and General Practice, Norwegian University of Science and Technology, Verdal, Norway; 7 The Cancer Clinic, Rikshospitalet-Radiumhospitalet Medical Center, University of Oslo, Oslo, Norway

## Abstract

**Background:**

The 2005 International Diabetes Federation (IDF) definition of the metabolic syndrome was designed to be useful worldwide, but to date few prevalence studies have used that definition in European populations. We estimated the age- and sex-stratified prevalence of IDF-defined metabolic syndrome in a county of Norway and compared it with the prevalence estimated using the revised National Cholesterol Education Program-Adult Treatment Panel-III definition (2005 ATP III).

**Methods:**

Cross-sectional analysis of 10,206 participants aged 20–89 years in the Nord-Trøndelag Health Study 1995–97 (HUNT 2).

**Results:**

Prevalence of IDF-defined metabolic syndrome was 29.6% (95% CI: 28.8 to 30.5), compared to 25.9% (95% CI: 25.0 to 26.7) using the 2005 ATP III criteria. The prevalence of IDF-defined metabolic syndrome increased from 11.0% in the 20–29 years age group to 47.2% in the 80–89 years group in men, and from 9.2% to 64.4% for women in the corresponding age groups. Among men and women aged ≥60 years, the IDF criteria classified 56.7% and 75.0%, respectively, as having central obesity, and 89.3% and 90.9%, respectively, as being hypertensive.

**Conclusion:**

According to both definitions, the prevalence of the metabolic syndrome increased strongly with age. The IDF and the American Heart Association/National Heart, Lung, and Blood Institute guidelines for clinical management of metabolic syndrome would classify a high proportion of elderly Norwegians as in need of overall risk assessment for cardiovascular disease.

## Background

The metabolic syndrome has been identified as a cluster of risk factors for atherosclerotic cardiovascular disease and type 2 diabetes, and its occurrence has been described as a global epidemic [1]. In the past few years, increased attention to the syndrome has led to several attempts to develop a definition accepted worldwide. In 1998, the World Health Organization (WHO) proposed a set of diagnostic criteria [2], followed by definitions from the European Group for the Study of Insulin Resistance (EGIR) in 1999 [3], and by the National Cholesterol Education Program's Adult Treatment Panel III (ATP III) in 2001 [4]. These definitions agreed that hyperglycemia, obesity, dyslipidaemia, and hypertension are core components of the metabolic syndrome, but they differed in the details and criteria. The 2001 ATP III definition has been regarded as probably the most practical for alerting clinicians to subjects at risk [5]. However, the cut-off values for obesity have been criticized for limited applicability in various ethnic groups [6]. In 2005, a modification (2005 ATP III) by the American Heart Association and National Heart, Lung, and Blood Institute (AHA/NHLBI) was published. The 2001 ATP III criteria were maintained except for a reduced threshold for hyperglycemia and some minor modifications [7].

In 2005, the International Diabetes Federation (IDF) issued new criteria that further modified the ATP III definition [6,8]. The IDF considered that visceral obesity is highly correlated with insulin resistance (which was a core factor in the WHO definition, but cumbersome to assess in clinical practice), and thus essential for diagnosis of the metabolic syndrome. The waist circumference thresholds were lower than the ATP III recommendations and were made ethnicity-specific in an effort to make the definition useful in any population. The IDF definition required central obesity plus any two of four components: elevated triglycerides, low high-density lipoprotein (HDL) cholesterol, hypertension and elevated plasma glucose.

The IDF recommended, "Management of the condition should be aggressive and uncompromising in its aim to reduce the risk of cardiovascular disease and type 2 diabetes. Patients should undergo a full cardiovascular risk assessment" [8]. Similarly, the 2005 AHA/NHLBI scientific statement recommended, "to reduce lifetime risk for atherosclerotic cardiovascular disease, all individuals found to have the metabolic syndrome deserve long-term management and follow-up in the clinical setting" [7]. Thus, information about the prevalence of metabolic syndrome is essential in order to estimate the health care resources needed for making the diagnosis, counselling about lifestyle changes, and doing overall risk assessment for cardiovascular disease. To date, the prevalence of metabolic syndrome defined by the IDF or the 2005 ATP III criteria is unknown in the general population in the northern part of Europe.

In this study, we estimated age- and sex-specific prevalence rates of the metabolic syndrome in the Norwegian population participating in the Nord-Trøndelag Health Study 1995–97 using the IDF definition, compared the rates with those obtained using the 2005 ATP III definition, and estimated the concordance between the two definitions.

## Methods

### Sample characteristics

Our cross-sectional data were derived from the Nord-Trøndelag Health Study (HUNT 2) 1995–97, a Norwegian population study which is described in detail elsewhere [9]. Briefly, all inhabitants aged 20 years and above in the county were invited to a general health study. Data were obtained from physical tests, blood samples, and from questionnaires that covered demographic characteristics, somatic illnesses, somatic and mental symptoms, medications, life style, and health-related behaviour. Among a total of 92,205 individuals aged 20 to 89 years, 65,753 (71.3%) participated, and 63,600 individuals had valid data for this study. As the HUNT study is a large survey where participants were examined at different time points during the day, it was impractical to keep everyone fasting. Blood sampling was done whenever the participants attended, but time since the last meal was noted. In all, 10,206 individuals (16% of the 63,600) reported ≥4 hours since their last meal, and they were included in the present study. Based on data from the fourth (2394) of the included sample that reported ≥9 hours fasting, triglycerides and glucose levels for individuals fasting 4–8 hours were adjusted before further analyses were performed.

In the HUNT 2 study, blood pressure was measured by specially trained nurses, using a Dinamap 845XT (Criticon, Tampa, Florida, USA) based on oscillometry. Cuff size was adjusted according to the arm circumference. The Dinamap was started after the subject had been seated for two minutes with the cuff on the arm, and the arm resting on a table. Blood pressure was measured three times at one-minute intervals. The mean of the second and third reading was used in this study.

Blood samples were analysed at Levanger Hospital on a Hitachi 911 autoanalyser. Glucose was measured with an enzymatic hexokinase method, total cholesterol and HDL cholesterol applying an enzymatic colorimetric cholesterolesterase method and triglycerides by an enzymatic colorimetric method. Participants with a history of diabetes were invited to an additional fasting blood test for glucose, C-peptide and glutamic acid decarboxylase antibodies. Based on these and information on start of insulin treatment, diabetes types 1 and 2 were identified.

### Definitions of the metabolic syndrome

The IDF definition [8] requires central obesity (measured as ethnic-specific waist circumference; for population of European origin: ≥94 cm in men and ≥80 cm in women) plus any two of four components: 1) serum triglycerides ≥1.7 mmol/L or specific treatment for this lipid abnormality; 2) HDL cholesterol <1.03 mmol/L in men and <1.29 mmol/L in women or specific treatment for this lipid abnormality; 3) blood pressure ≥130/≥85 or treatment for previously diagnosed hypertension; and 4) fasting plasma glucose ≥5.6 mmol/L or previously diagnosed type 2 diabetes. Data on specific treatment for lipid abnormalities were not available in the HUNT 2 study; however, such treatment was infrequent in Norway at that time.

The 2005 ATP III definition [7] requires the presence of three or more of five components: 1) waist circumference >102 cm in men and >88 cm in women, 2) triglycerides ≥1.7 mmol/L or drug treatment for elevated triglycerides, 2) HDL cholesterol <1.03 mmol/L in men and <1.29 mmol/L in women or drug treatment for reduced HDL cholesterol, 4) blood pressure ≥130/≥85 mm Hg or drug treatment for previously diagnosed hypertension, and 5) fasting plasma glucose ≥5.6 mmol/L or drug treatment for elevated glucose.

### Statistical analysis

Descriptive statistics were used to estimate age and the components of the metabolic syndrome in the 10,206 participants fasting ≥4 hours (the included sample) in relation to sex and the IDF defined metabolic syndrome. Distribution (means with 95% confidence interval, CI) of plasma glucose, triglycerides, and HDL cholesterol in relation to time since the last meal were explored with graphs and regression analyses.

After more than four hours fasting, we found no statistical significant association between time since the last meal and HDL cholesterol. However, triglycerides were weakly and negatively associated with time since the last meal (0.042 mmol/L decrease per hour fasting from 4 to 9), whereas glucose was weakly and positively associated (0.061 mmol/L increase per hour fasting from 4 to 9). We adjusted the triglyceride and glucose levels for time since the last meal based on regression coefficients obtained from linear regression models with triglycerides and glucose, respectively, as the dependent variable, and time since the last meal (range 4–9 hours) as independent variable (encoded categorically), all models adjusted for age (continuous measure) and sex. We regarded individuals reporting ≥9 hours since the last meal as fasting and used their triglyceride and glucose levels as references for the adjustments. Triglyceride levels were subtracted 0.208, 0.135, 0.044, 0.076, and 0.014 mmol/L for individuals reporting fasting for 4, 5, 6, 7, and 8 hours, respectively. Correspondingly, glucose levels were added 0.307, 0.284, 0.216, 0.272, and 0.006 mmol/L.

We calculated prevalence estimates of IDF-defined metabolic syndrome for the included sample, for men and women, and for sex-stratified 10-years age groups. Sex-stratified prevalence rates of the other components of IDF-defined metabolic syndrome in relation to central obesity were estimated. Prevalence of the metabolic syndrome estimated by the IDF and the 2005 ATP III definitions was presented as age- and sex-stratified graphs, and concordance between the two definitions was estimated by classification in percentages, and by κ statistic.

Finally, for the purpose of examining the representativeness of the included sample, we estimated sex-stratified means and proportions (with 95% CI) of age and the components of metabolic syndrome in the 10,206 participants fasting ≥4 hours versus the 53,394 participants fasting <4 hours. We compared prevalence of metabolic syndrome in the two samples by excluding glucose and triglycerides from the IDF and 2005 ATP III definitions, with that minimizing the effects of fasting. Accordingly, the IDF-proxy for metabolic syndrome was defined as central obesity plus any two of three components (low HDL cholesterol, hypertension, or type 2 diabetes), and the ATP-proxy as any three of four components (central obesity, low HDL cholesterol, hypertension, or type 2 diabetes). The prevalence of the two proxies was calculated. Logistic regression analyses were employed (with fasting ≥4 hours as a dichotomous independent variable) for categorical variables and a *t *test for z-scores of continuous variables (See Results, last paragraph).

Two-sided P < 0.05 indicated statistical significance. All analyses were done with SPSS software (version 14.0; SPSS Inc, USA).

### Ethics

All participants in the HUNT study gave written informed consent. The Norwegian Data Inspectorate and the Regional Committee for Medical Research Ethics approved the study.

## Results

Characteristics of the included men and women with and without IDF-defined metabolic syndrome are shown in Table 1.

**Table 1:** Characteristics of participants^1 ^in relation to IDF-defined metabolic syndrome, the HUNT 2 study

	Men		Women	
	No metabolic syndrome	Metabolic syndrome	No metabolic syndrome	Metabolic syndrome

N (%)	3622 (71.1)	1479 (29.0)	3558 (69.7)	1547 (30.3)
Age, y	45.8 ± 16.6	55.0 ± 16.3	44.8 ± 16.1	60.2 ± 16.2
Systolic BP, mm Hg	136.9 ± 18.7	148.8 ± 19.9	130.1 ± 21.1	153.6 ± 24.3
Diastolic BP, mm Hg	81.2 ± 11.7	88.8 ± 11.7	77.1 ± 11.2	86.7 ± 12.9
Waist circumference, cm	88.2 ± 7.9	101.9 ± 7.3	77.4 ± 9.7	92.9 ± 9.9
HDL cholesterol, mmol/L	1.32 ± 0.33	1.10 ± 0.31	1.59 ± 0.37	1.30 ± 0.36
Triglycerides, mmol/L	1.31 ± 0.91	2.30 ± 1.40	0.99 ± 0.55	2.02 ± 1.15
Glucose, mmol/L	5.35 ± 0.93	5.93 ± 1.67	5.18 ± 0.60	5.98 ± 1.52
Type 2 diabetes, %	0.8	3.6	0.2	6.0
Antihypertensive medication, %	6.6	19.4	5.6	30.2
Waist/hip ratio	0.88 ± 0.05	0.95 ± 0.05	0.78 ± 0.05	0.85 ± 0.06
BMI, kg/m^2^	25.4 ± 3.0	29.8 ± 3.3	25.0 ± 4.1	30.2 ± 4.5
Total cholesterol, mmol/L	5.74 ± 1.17	6.25 ± 1.14	5.75 ± 1.29	6.69 ± 1.37

Data are numbers, means ± standard deviation, or percentages.

HUNT, the Nord-Trøndelag Health study; IDF, the International Diabetes Federation; BP, blood pressure; HDL, high-density lipoprotein; BMI, body mass index.

^1^Fasting ≥4 hours.

### IDF-defined metabolic syndrome

The prevalence of IDF-defined metabolic syndrome was 29.6% (95% CI: 28.8 to 30.5). This was similar in men (29.0%, 95% CI: 27.7 to 30.2) and in women (30.3%, 95% CI: 29.0 to 31.6). The prevalence increased with age right up to the ninth decade, especially among women (Table 2).

**Table 2:** Prevalence of IDF-defined metabolic syndrome and its components in sex and age groups

		IDF-defined metabolic syndrome	Obligate criterion	Additional criteria			
			
			Central obesity^1^	Hyper-triglyceridaemia^2^	Low HDL chol-esterol^3^	High blood pressure^4 ^or medication use	High plasma glucose^5 ^or type 2 diabetes
	N	% (95% CI)	%	%	%	%	%

*Men*							
20–29 yr	808	11.0 (8.9 – 13.2)	20.8	19.8	28.5	59.8	13.6
30–39 yr	966	20.9 (18.3 – 23.5)	34.1	29.9	27.4	58.5	22.8
40–49 yr	1065	27.6 (24.9 – 30.3)	40.9	38.7	30.9	69.4	28.8
50–59 yr	848	32.7 (29.5 – 35.8)	48.2	35.1	24.5	77.9	37.4
60–69 yr	665	41.7 (37.9 – 45.4)	55.2	37.0	26.9	87.1	46.9
70–79 yr	569	44.8 (40.7 – 48.9)	56.8	36.6	31.1	90.7	52.9
80–89 yr	180	47.2 (39.9 – 54.5)	62.2	40.0	31.1	93.3	57.2
20–89 yr	5101	29.0 (27.7 – 30.2)	42.0	33.3	28.3	72.8	32.7
*Women*							
20–29 yr	807	9.2 (7.2 – 11.2)	30.5	7.6	28.4	21.8	8.6
30–39 yr	877	14.1 (11.8 – 16.4)	39.0	10.6	27.5	29.4	14.1
40–49 yr	1014	21.8 (19.3 – 24.3)	48.7	17.3	25.7	47.7	21.1
50–59 yr	862	30.0 (27.0 – 33.1)	56.5	22.5	21.3	70.5	30.2
60–69 yr	645	49.0 (45.1 – 52.9)	70.7	34.7	26.7	86.7	45.6
70–79 yr	661	60.4 (56.6 – 64.1)	77.8	40.5	31.3	94.1	52.6
80–89 yr	239	64.4 (58.4 – 70.5)	78.7	44.4	38.1	93.7	58.6
20–89 yr	5105	30.3 (29.0 – 31.6)	53.4	22.0	27.1	57.4	28.4

IDF, the International Diabetes Federation; HDL, high-density lipoprotein; CI, confidence interval.

^1^Waist circumference ≥94 cm in men, ≥80 cm in women.

^2^Triglycerides ≥1.7 mmol/L.

^3^HDL cholesterol <1.03 mmol/L in men, <1.29 mmol/L in women.

^4^Systolic blood pressure ≥130 mm Hg or diastolic blood pressure ≥85 mm Hg.

^5^Plasma glucose ≥5.6 mmol/L.

The prevalence of IDF-defined central obesity was 42.0% among men and 53.4% among women. Among men and women aged 60 years or more, the IDF criteria classified 56.7% and 75.0%, respectively, as having central obesity, and 89.3% and 90.9%, respectively, as hypertensive.

In men with central obesity, 83.3% had high blood pressure or used antihypertensive medications, 46.9% had high triglycerides, 40.2% had high glucose or type 2 diabetes, and 38.0% had low HDL cholesterol, by the IDF criteria. In women, the corresponding percentages were 72.3%, 34.1%, 37.2% and 36.1%. In total, 69.0% of the men and 56.7% of the women with central obesity had at least two additional components defining the metabolic syndrome. Central obesity without any additional component was infrequent (5.9% in men and 12.6% in women).

### IDF versus 2005 ATP III

**Figure 1:**
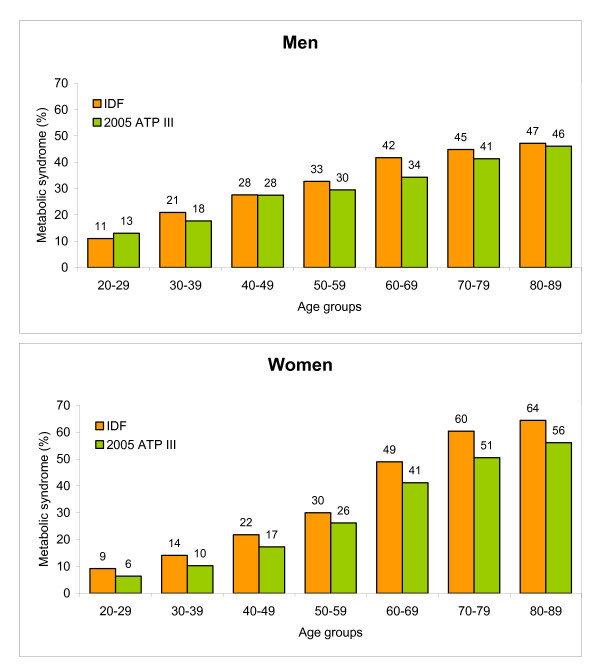
Age-specific prevalence of metabolic syndrome according to the definition by the International Diabetes Federation (IDF), compared to the revised definition by the National Cholesterol Education Program-Adult Treatment Panel III (2005 ATP III), among men and women in the HUNT 2 population.

The IDF definition of the metabolic syndrome gave a higher prevalence in all demographic groups except in men aged 20–29 years, compared to estimates by the 2005 ATP III criteria (Figure 1). In the entire study sample, the prevalence of the syndrome was 25.9% (95% CI: 25.0 to 26.7) by the 2005 ATP III criteria; the prevalence was 26.8% in men and 25.0% in women.

By applying either the IDF or the 2005 ATP III criteria, 34.5% of the participants were defined as having the metabolic syndrome (21.0% by both criteria, 8.6% by IDF only, and 4.9% by 2005 ATP III only), and 65.5% as not having the metabolic syndrome, providing a concordant classification for 86.5% of the participants. Overall, agreement between the IDF and the 2005 ATP III criteria was good (total κ 0.66, men κ 0.57, women κ 0.76). Although κ showed some variations between sex-specific 10-year age groups, there were no age trends.

According to the higher threshold for central obesity in the 2005 ATP III criteria, the prevalence of obesity was only one third (13.5%) in men and one half (27.2%) in women as compared to the prevalence obtained by the IDF criteria.

### Representativeness of the included sample

There were minor differences between the fasting (the included sample) and the non-fasting participants in the HUNT 2 study (Table 3). Fasting participants were on the average one year younger than non-fasting participants. For some of the components of metabolic syndrome, we found statistically significant differences in one or both sexes. Although statistically significant, these differences were minor, except for the glucose and triglyceride levels that differed somewhat more, as expected.

**Table 3:** Characteristics of fasting^1 ^versus non-fasting participants by sex, the HUNT 2 study

	Men			Women		
	Fasting^1^	Non fasting		Fasting^1^	Non fasting	

N (%)	5101 (17.0%)	24,914 (83.0%)		5105 (15.2%)	28,480 (84.8%)	

*Categorical measures, % (95% CI)*			*Odds ratio*			*Odds ratio*

IDF-proxy for metabolic syndrome^2^	13.8 (12.8–14.7)	14.4 (14.0–14.9)	1.05 (0.97–1.15)	14.8 (13.9–15.8)	13.8 (13.4–14.2)	0.92 (0.85–1.00)
ATP-proxy for metabolic syndrome^3^	5.8 (5.2–6.5)	5.8 (5.5–6.1)	1.00 (0.88–1.14)	9.8 (8.9–10.6)	9.0 (8.7–9.4)	0.92 (0.83–1.02)
Type 2 diabetes	1.6 (1.2–1.9)	1.9 (1.7–2.0)	1.18 (0.93–1.49)	2.0 (1.6–2.4)	1.6 (1.5–1.8)	0.81 (0.65–1.01)
Antihypertensive medication	10.3 (9.5–11.1)	10.3 (9.9–10.6)	1.00 (0.90–1.10)	13.1 (12.2–14.0)	11.6 (11.2–12.0)	0.87* (0.80–0.96)

*Continuous measures, means (95% CI)*			*Mean z-score diff.*			*Mean z-score diff.*

Age, y	48.4 (48.0–48.9)	50.0 (49.8–50.2)	0.093*	49.4 (48.9–49.8)	50.2 (50.0–50.4)	0.048*
Systolic blood pressure, mm Hg	140.4 (139.8–140.9)	140.0 (139.7–140.2)	-0.022	137.3 (136.6–137.9)	135.8 (135.5–136.1)	-0.062*
Diastolic blood pressure, mm Hg	83.4 (83.0–83.7)	81.7 (81.6–81.9)	-0.138*	80.0 (79.6–80.3)	78.7 (78.6–78.9)	-0.101*
Waist circumference, cm	92.2 (91.9–92.5)	91.9 (91.8–92.0)	-0.028	82.0 (81.7–82.4)	81.5 (81.4–81.6)	-0.050*
HDL cholesterol, mmol/L	1.26 (1.25–1.27)	1.24 (1.23–1.24)	-0.063*	1.50 (1.49–1.52)	1.49 (1.49–1.50)	-0.029
Triglycerides, mmol/L	1.73 (1.70–1.76)	2.02 (2.01–2.04)	0.240*	1.44 (1.42–1.47)	1.59 (1.58–1.60)	0.153*
Glucose, mmol/L	5.30 (5.27–5.33)	5.60 (5.58–5.62)	0.185*	5.20 (5.17–5.23)	5.41 (5.40–5.43)	0.150*

HUNT, the Nord-Trøndelag Health study; CI, confidence interval; IDF, the International Diabetes Federation; ATP, the National Cholesterol Education

Program-Adult Treatment Panel III; HDL, high-density lipoprotein.

*P < 0.05.

^1^Four to nine hours or more since the last meal.

^2^IDF-proxy for metabolic syndrome defined as IDF-central obesity plus any two of three components (low HDL cholesterol, high blood pressure or treatment for previously diagnosed hypertension, or previously diagnosed type 2 diabetes).

^3^ATP-proxy for metabolic syndrome defined as any three of four components (ATP III-defined central obesity, low HDL cholesterol, high blood pressure or treatment for previously diagnosed hypertension, or previously diagnosed type 2 diabetes).

We found no statistically significant difference between the fasting and the non-fasting samples in the prevalence of the IDF- or ATP-proxies of metabolic syndrome, neither in sex-stratified analyses (Table 3) nor in analyses stratified by sex and 10-year age groups (data not shown).

## Discussion

Applying the IDF definition in a large Norwegian population aged 20–89 years classified 29.6% as having the metabolic syndrome. The prevalence was highly age-dependent. This was evident especially in women, with a sevenfold increase in prevalence from age group 20–29 years to age group 80–89 years.

The IDF emphasizes central obesity as an essential criterion for the metabolic syndrome, due to the evidence linking ethnicity-specific waist circumference to cardiovascular disease and to the other components of the metabolic syndrome [6]. Applying the recommended cut-offs for white people of European origin classified a large portion of this Norwegian population as having central obesity. In women aged 60 years or more, three out of four were defined as obese. As central obesity is regarded as a likely early step in the development of full metabolic syndrome [6,10], this definition puts a very large number of individuals belonging to one of the longest living and healthiest populations in the world [11] at increased risk for cardiovascular disease and type 2 diabetes.

The IDF definition classified the majority of our population as hypertensive. Mean blood pressure in the HUNT 2 population increased markedly with age, especially among women. As the IDF definition, like other definitions of the metabolic syndrome, has not included any adjustment for age, 90% of those aged 60 years or more would be defined as being hypertensive.

The prevalence of the metabolic syndrome by the IDF criteria was higher than by the 2005 ATP III criteria in all sex-stratified age groups except in men 20–29 years old. As the two definitions are based on much of the same components, the difference in prevalence was mainly related to different waist circumference and to the focus on central obesity as an obligatory component in the IDF definition in contrast to being one out of five equally weighted components in the 2005 ATP III definition.

Several population studies have reported an increase in the prevalence of the metabolic syndrome with age regardless of definition [12-31], although some have reported a peak in the seventh decade and then a decline in both sexes [12,19,23,31] or only in men [13,14,18,22,26,30]. However, this has been incompletely studied in European populations, where only one study from Greece has assessed age-specific prevalence using the IDF definition [23]. In addition, to our knowledge, only one study worldwide has previously assessed the prevalence separately for those aged 80 years and above [31]. In contrast to several other studies [12-14,18,19,22,23,26,31], we found that the prevalence of the metabolic syndrome, by both definitions, increased linearly with age beyond the seventh decade in both sexes. To our knowledge, our study is the first to show that this increase continued into the ninth decade.

### Strengths and limitations

The HUNT 2 study covered the whole adult population (homogenous, <3% non-Caucasian) in Nord-Trøndelag County, which is considered fairly representative of Norway. The county is mostly rural; the largest of six small towns has a population of 21,000. The main objectives of the HUNT study concerned large public health issues like diabetes, cardiovascular disease, obstructive lung disease, osteoporosis and mental health [9].

The participation rate was age dependent, with the highest participation rate (85.6%) in the age group 60–69 years. A study of non-participants [9] showed that the main reasons for not attending were of a practical nature in young people and poor health in elderly people. There is a potential selection bias from non-participation, although the participation rate in the HUNT study was similar to or higher than in comparable population studies [9].

A limitation of the HUNT study is that it was impractical to request the whole population to be fasting since examinations were spread out during daytime. Blood sampling was done whenever participants attended. However, our estimates of prevalence of the metabolic syndrome based on the sub-sample of participants reporting ≥4 hours fasting should be quite representative for the entire HUNT 2 population as the statistically significant differences between fasting and non-fasting participants were all minor (except triglycerides and glucose). Further, using the proxies of IDF and ATP metabolic syndrome, we were unable to reveal any statistically significant difference between fasting and non-fasting participants. Our adjustment of the levels of plasma glucose and triglycerides among the included individuals reporting 4–8 hours fasting should further increase the validity of our findings. Other referenced prevalence studies have used 6 hours fasting without adjustments [15,31,32].

### Management of metabolic syndrome

The IDF Epidemiology Task Force Consensus Group [6] states, "Researchers and clinicians should use the new criteria for the identification of high-risk individuals and for research studies. Preventive measures are obviously needed in people identified". According to the AHA/NHLBI Scientific Statement 2005, the prime emphasis in individuals with metabolic syndrome is to mitigate the underlying risk factors (obesity, physical inactivity, and atherogenic diet) through lifestyle changes [7]. In addition, 10-year risk assessment (e.g. age, gender, total cholesterol, smoking status) for cardiovascular disease should be carried out with algorithms such as the Framingham risk score [1,7,33].

All components of the IDF criteria can easily be measured in primary care and used as tools for counselling. However, when the majority of elderly individuals are defined as being in need of risk management, this becomes a challenge both for clinicians and researchers. It seems ethical questionable to identify such a large part of the population as being at risk, particularly since there still is doubt regarding the value of the metabolic syndrome as a risk marker. On the one hand, data indicate that the metabolic syndrome carries increased long-term risk both for cardiovascular disease and diabetes as well as a short-term risk [33]. On the other hand, the meaning of the metabolic syndrome as a cardiovascular risk factor independent of other conventional risk factors (e.g., smoking, family history) has been questioned [34]. In addition, few studies have examined whether this risk factor is as strong in elderly individuals as it is in younger or middle-aged individuals. A recent study among Swedish men followed for a maximum of 33 years found that the metabolic syndrome was an independent risk factor in middle age but not consistently in elderly men for cardiovascular and total mortality, when established cardiovascular risk factors were taken into account [35]. Other studies among elderly people have not been conclusive: in British women, the syndrome was found only modestly associated with risk of coronary heart events in un-adjusted analyses, and not associated when adjusted for other risk factors [32], and in American men and women, the syndrome was found associated with increased cardiovascular events but not with total mortality [36].

Thus, implementation of the recent IDF and AHA/NHLBI guidelines for clinical management of the metabolic syndrome in elderly individuals may seem somewhat premature. First, further data are needed to assess the risk for cardiovascular and total mortality. Secondly, as the metabolic syndrome is not a reliable tool for overall risk assessment in the short term but more carries increased long-term risk [33], this may be of lower value among elderly than among younger people. Thirdly, our findings should give increased attention to the practical, ethical, and economic aspects of classifying a very high portion of asymptomatic elderly individuals as in need of counselling, overall risk assessment for cardiovascular disease, and long-term follow-up.

## Conclusion

We found a high prevalence of the metabolic syndrome in Norwegian adults aged 20–89 years, particularly by using the IDF criteria. Our findings of increasing prevalence right up to the ninth decade highlight the need for further studies to assess if the associated risks are the same in elderly people as what have been found in younger people. Hopefully, this will clarify if the metabolic syndrome is a meaningful diagnosis in elderly people.

## Competing interests

The author(s) declare that they have no competing interests.

## Authors' contributions

All authors participated in the study design, evaluation of data and discussions of the draft outline, and contributed later with text revisions, table revisions and figure revisions. AAD conceived the study. BH planned the study, performed the analyses and drafted the manuscript. AM supervised the statistical analysis. TH and KM contributed with their knowledge on cardiology and diabetes, respectively. KM contributed with his knowledge of how the HUNT study was carried out. All authors read and approved the final manuscript.

## Pre-publication history

The pre-publication history for this paper can be accessed here:


